# A Novel Sensor for Undrained Shear Strength Measurement in Very Soft to Soft Marine Sediments Based on Optical Frequency Domain Reflectometry Technology

**DOI:** 10.3390/s22155530

**Published:** 2022-07-25

**Authors:** Pei-Chen Wu, Wen-Bo Chen, Jian-Hua Yin, Yu Pan, Kai Lou, Wei-Qiang Feng

**Affiliations:** 1 Department of Civil and Environmental Engineering, The Hong Kong Polytechnic University, Hong Kong, China; peichen.wu@connect.polyu.hk (P.-C.W.); geocwb@gmail.com (W.-B.C.); cejhyin@polyu.edu.hk (J.-H.Y.); yu1994.pan@connect.polyu.hk (Y.P.); kai.lou@connect.polyu.hk (K.L.); 2 Department of Civil and Environmental Engineering, Research Institute of Land and Space, The Hong Kong Polytechnic University, Hong Kong, China; 3 Department of Ocean Science and Engineering, Southern University of Science and Technology, Shenzhen 518055, China; 4 Southern Marine Science and Engineering Guangdong Laboratory (Guangzhou), Guangzhou 510000, China

**Keywords:** marine soil, undrained shear strength, optical fiber sensing, OFDR technology

## Abstract

Due to the short supply of conventional fill materials, such as sand, land reclamation using dredged marine deposits has recently been proposed, in which marine deposits with high water content are blow-filled into reclaiming areas. The strength development of the filled marine soils is of great importance during the sedimentation and consolidation to guide the filling process and construction of reclamation. In this study, a novel sensor based on optical frequency domain reflectometry (OFDR) technology with a simple design was developed for undrained shear strength measurement. The novel sensor consists of an optical fiber and a series of polyoxymethylene coins. Owing to the merits of OFDR technology on high resolution, fully distributed sensing, and immunity to electromagnetic interference, the novel sensor can be used to determine undrained shear strength profiles of very soft to soft marine sediments/soils with good accuracy. The sensor was calibrated in remolded marine deposits with different water contents. The good feasibility and performance of the novel sensor for undrained shear strength measurement were well validated in two physical model tests on marine deposits treated by horizontal drains with vacuum preloading.

## 1. Introduction

To solve the issue of the short supply of lands for buildings and infrastructures, marine reclamations have a long history in Hong Kong and other coastal cities around the world. The technologies used for reclamations vary widely. The typical method is to fill reclaimed areas with conventional granular fill materials, such as sand or crushed stones. For coastal cities, such as Singapore and Hong Kong, filling materials are mainly imported, extremely increasing the construction cost and time. Owing to a shortage of sand fill, dredged marine deposits have been recently utilized for reclamation using blow-filling or dumping method in Tianjin of China [1] and Singapore [2], and will be of great potential to Hong Kong [3]. Using the dredged marine soil as filling materials is challenging because of the low shear strength and potential excessive post-construction settlements. Among various ground improvement methods, a method using horizontal drains with vacuum preloading was found to be the most efficient way to improve the dredged marine soils [4,5]. In this method, dredged soils with high water content or slurry are blow-filled in stages. Due to the high water content, sedimentation and self-weight consolidation usually take place before the application of vacuum preloading [6,7]. Strength development of the blow-filled soils is of great importance to guide the filling process of reclamation projects and to assess the efficiency of ground improvement techniques. In addition, to assess the embedment of a subsea pipeline during laying and the interaction forces generated as the pipe moves across the seabed, it is necessary to determine the strength of the upper 0.5 m of sediments with good resolution [8]. Therefore, a proper method for measuring the strength profiles of very soft to soft seabed soils, such as blow-filled soil or near-surface sediments, is essential in geotechnical engineering and offshore constructions.

Strength profiles of soil are commonly measured in situ by conducting a cone penetrometer test (CPT) and a vane shear test (VST). Recently, T-bar or ball penetrometers have been used to measure the strength of sediments [9,10]. T-bar and ball penetrometers have advantages over conventional CPT and VST, including (i) improving the resolution of the measured bearing resistance due to a greater bearing area; (ii) creating an exact solution to convert bearing resistance to shear strength due to the well-defined failure mechanism; and (iii) suitable for both static and cyclic shear strength measurement [11,12]. In order to accurately measure soil strength, there is a certain restriction on the size of the T-bar. In addition, T-bar penetrometers are susceptible to bending moments, which may affect the measurement of bearing resistance [13]. Except for VST, the aforementioned methods for determining shear strength rely on the bearing resistance measured by load cells, whose resolution, quality, and reliability affect the measurands. Table 1 summarizes the disadvantages of typical CPT, VST, and T-bar/ball penetrometers.

Optical fiber sensing technologies, such as fiber Bragg grating (FBG) sensing, Brillouin-based distributed sensing, and optical frequency domain reflectometry (OFDR) sensing, have been widely used in geotechnical engineering because of their merits in quasi or fully distributed sensing, real-time monitoring, high accuracy and stability, immunity to electromagnetic interference, and capacity of multiplexing [14,15,16,17,18,19,20]. Compared to FBG and Brillouin-based distributed sensing technologies, OFDR sensing technology usually has a higher spatial resolution. In this study, a simple and novel sensor based on OFDR technology was designed for measuring the undrained shear strength profile of very soft to soft sediments/soils. The proposed sensor was first calibrated in remolded marine deposits with different water contents and then adopted in two physical model tests with two different setups. Horizontal drains were installed at the bottom of the two physical model tests. Vacuum preloading was applied through the horizontal drains to consolidate the soil inside the physical models.

## 2. Measurement Principle

**Figure 1:**
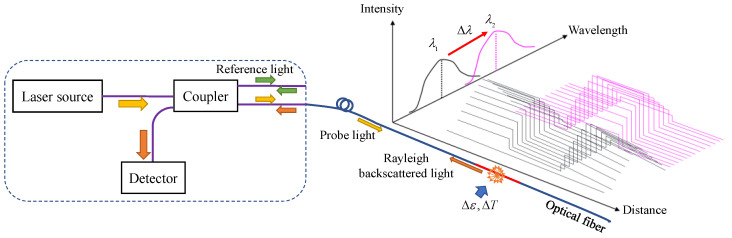
Measuring principle of OFDR technology.

OFDR technology can be used to measure strain, temperature, and vibration. The principle of strain and temperature measurement using OFDR technology is illustrated in Figure 1. The interrogator of OFDR mainly consists of a linear sweep laser source, a coupler, and a detector. The coupler splits the incident light into a reference light and a probe light that transmits in optical fibers. Owing to the intrinsic material heterogeneity and density fluctuation of the transmit or sensing optical fiber, Rayleigh scattering of the probe light occurs along the fiber, in which the scattered light with the direction opposite to the probe light is guided back to the coupler. A beat frequency interference of the scattered light and the reflected reference light is produced by the coupler and analyzed by the detector [21,22]. The spectrum of the scattered light is a function of distance along the fiber, thereby achieving distributed sensing. Changes in strain and temperature induce shifts of scattered light spectrum. The relationship between the Rayleigh frequency shift and strain and temperature changes can be described by the following equation [19,23]:

(1) $$\Delta \nu =C_{\varepsilon }\Delta \varepsilon +C_{T}\Delta T$$

where Δν refers to the Rayleigh frequency shift, and $C_{\varepsilon }$ and $C_{T}$ are the strain and temperature coefficients. A commercially available interrogator (Model: OSI-S, Megasense Technologies Co., Ltd., Wuhan, China) was used. The same PVC-coated single mode optical fibers (manufactured by YOFC Ltd., Wuhan, China) used by Hong et al. [24] and Feng et al. [19] were adopted in this study. The specification of the OFDR interrogator and optical fibers is listed in Table 2. Although OFDR technology has been used for dynamic measurement [25], the OFDR interrogator used in this study is for static measurement only with a measuring rate of 1/3 Hz.

## 3. Sensor Design and Working Principles

**Figure 2:**
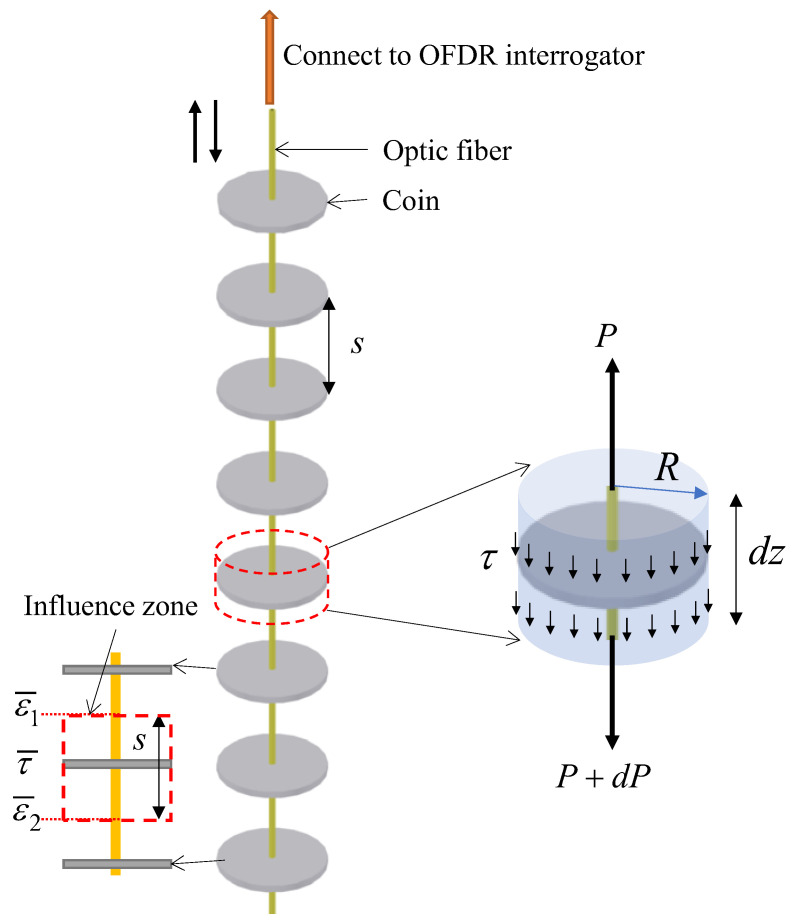
Illustration of the working principle of the OFDR coin sensor.

Figure 2 illustrates the design of the novel sensor for undrained shear strength measurement. The sensor consists of an optical fiber and a series of polyoxymethylene (POM) coins with the radius of *R* and the spacing of *s*. The sensor can be pre-installed or inserted when required into the testing soil. During the measurement, the optical fiber is pulled up or down. It is assumed that a cylindrical shear surface with the radius equal to that of the coin exists where shear stress is mobilized. Taking a cylinder unit as an example, the equilibrium in the vertical direction is expressed as:

(2) P+dP=P+2πRτdz

where P is the tension along the optical fiber, dP is the tension increment, R is the radius of the cylinder shear surface, τ is the shear stress on the shear surface, and dz is the thickness of the cylinder unit. The interfacial friction between the optical fiber and the surrounding soil is ignored because of the small contact area between the fiber and the very soft to soft sediments/soils of interest as well as the smooth nature of the fiber surface. Assuming the optical fiber is linear elastic, the tension P at a given point along the optical fiber can be determined considering the linear relationship between stress and strain [26]:

(3) $$P=A_{f}\sigma_{t}=E_{f}A_{f}\varepsilon$$

where $E_{f}$ and $A_{f}$ are the elastic modulus and cross-section area of the optical fiber; and $\sigma_{t}$ and ε are the tensile stress and the strain at the given point, respectively. The strain unit for the optical fiber is 1 micron per meter (10^−6^ m/m), which is indicated as 1 με. Combining Equations (2) and (3), the shear stress τ can be determined:

(4) $$\tau =\frac{E_{f}A_{f}}{2\pi R}\frac{d\varepsilon }{dz}$$

Equation (4) represents the working principle of this sensor, which relates the shear stress to the measurement of strain changes. It should be pointed out that this equation is valid only if there is a cylindrical shear surface. The real shape of the shear surface might not be cylindrical. Therefore, a factor α is introduced to correct the shape of the shear surface, which varies with the soil properties. The value of α can be determined through calibration tests. The change of strain along the optical fiber can be measured by OFDR technology. It can be seen from Equation (4) that the resolution of shear stress is inversely proportional to the radius of the coin as $E_{f}$ and $A_{f}$ are constant for a given optical fiber and the spatial resolution of the strain depends on the OFDR technology. Equation (4) does not take into account the elastic modulus of the soil within each sensing element (between two adjacent coins). The contribution of the modulus of the soil increases with the decrease in water content or increase in soil density. Since the OFDR coin sensors are designed for the application in very soft to soft sediments/soils, the contribution of the soil modulus is not significant. In addition, the normal stresses acting on the coins are not considered, as they are within the sensing element.

If the average strain between the adjacent coins is used, the average shear stress in the influence zone of a coin of interest can be expressed as:

(5) $$\bar{\tau }=\alpha \frac{E_{f}A_{f}}{2\pi Rs}\Delta \bar{\varepsilon }$$

where s is the spacing of two adjacent coins; $\Delta \bar{\varepsilon }={\bar{\varepsilon }}_{1}-{\bar{\varepsilon }}_{2}$. ${\bar{\varepsilon }}_{1}$ and ${\bar{\varepsilon }}_{2}$ are the average strains, as shown in Figure 2. The average strain can be calculated from the distribution profile of strain along with the fiber within each sensing element.

The resolution of the average shear stress is inversely proportional to the radius and spacing of the coins. However, increasing the spacing does not guarantee a high resolution of shear stress as the shear surface may not be formed in the case of larger spacing.

It should be noted that the influence of temperature on OFDR coin sensors can be ignored in the laboratory condition. However, temperature compensation is needed by the addition section of strain-free optical fiber for the temperature change only if the sensors are adopted for in situ measurement.

## 4. Calibration Tests

### 4.1. Setup and Preparation

**Figure 3:**
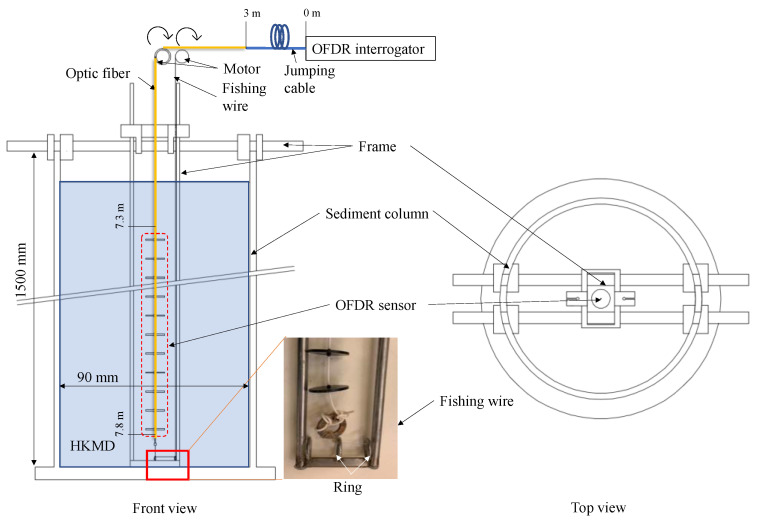
Sedimentation column for calibration tests.

To determine the factor α, the OFDR coin sensors were calibrated in the soil with different water contents. Equation (5) is rewritten as Equation (6):

(6) $$\bar{\tau }=k\Delta \bar{\varepsilon }$$

where $k=\alpha \frac{E_{f}A_{f}}{2\pi Rs}$. The calibration tests were conducted in a sedimentation column with the dimension of 90 mm in diameter and 1500 mm in height. A frame was designed to facilitate the installation and operation of the sensors, as shown in Figure 3. One end of the OFDR coin sensor was tied to a fishing wire that passed through two rings welded on the bottom of the frame. The other end of the OFDR coin sensor was connected to the OFDR interrogator using a 3 m jumping cable. The location of the interrogator (starting point of the jumping cable) was 0 m. The coins were located 7.3 m to 7.8 m along the entire cable. The OFDR coin sensor was installed in the sedimentation column together with the frame. The optical fiber and the fishing wire were let out from the top of the sedimentation column and wound to two motors, respectively. One motor was to pull the optical fiber, while the other was to pull the fishing wire. The sedimentation column was filled with remolded Hong Kong marine deposits (HKMD) with different water contents. The basic properties of HKMD are listed in Table 3. Shells were removed from the HKMD before mixing with water to reach a slurry state with the water contents (*wc*) of 110%, 150%, and 200% (around 2.5 to 4.5 times the liquid limit of the HKMD), respectively. It should be noted that ensuring a uniformly remolded HKMD slurry is difficult if the water content is less than 2 times the liquid limit. The calibration tests were conducted right after the filling of HKMD slurry to eliminate the significant density change with depth due to the self-weight consolidation. During the calibration tests, the OFDR sensor was pulled up by winching the optical fiber or pulled down by winching the fishing wire. A constant pulling rate of 0.5 mm/s was adopted for all calibration tests.

### 4.2. Undrained Shear Strength Measurement

**Figure 4:**
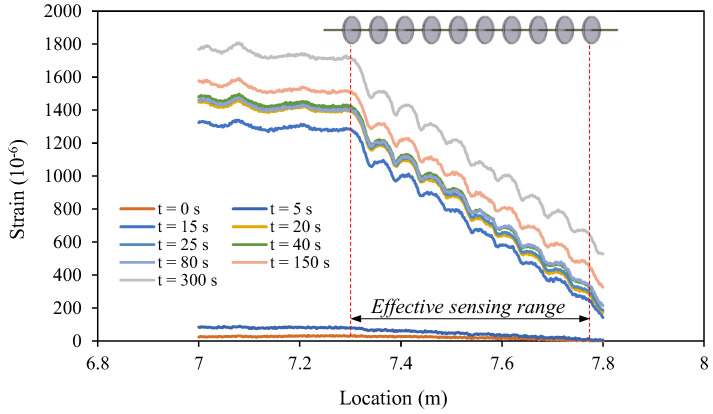
Typical strain distributions along the OFDR coin sensor.

**Figure 5:**
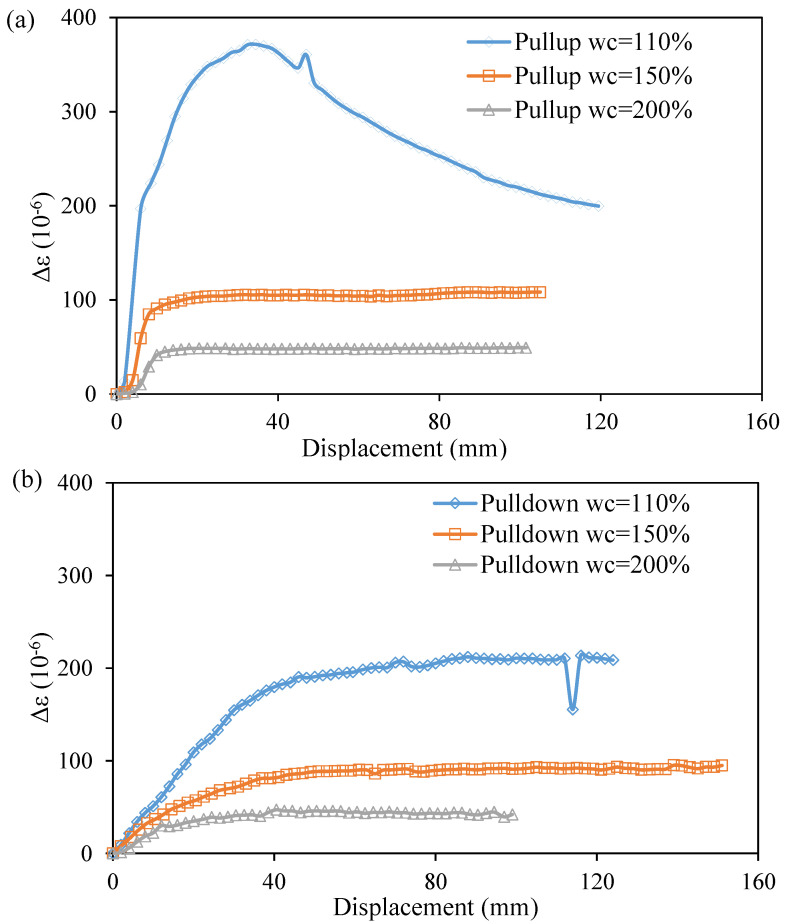
Measured strain change by the OFDR coin sensor: (**a**) pullup tests; and (**b**) pulldown tests.

**Figure 6:**
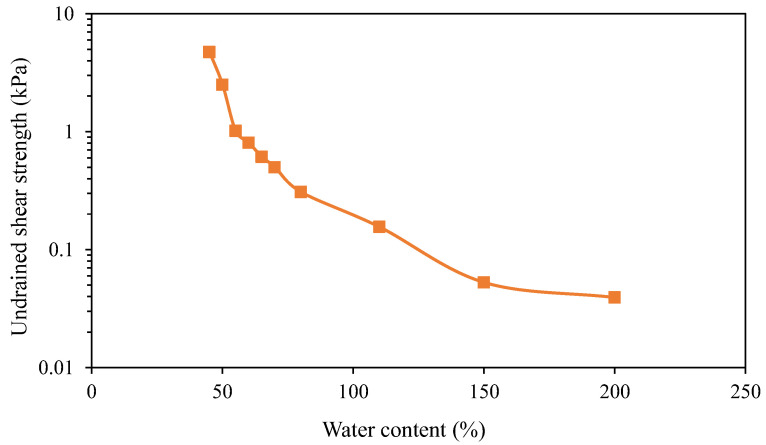
Reference undrained shear strength of HKMD with different water contents obtained from vane shear tests.

**Figure 7:**
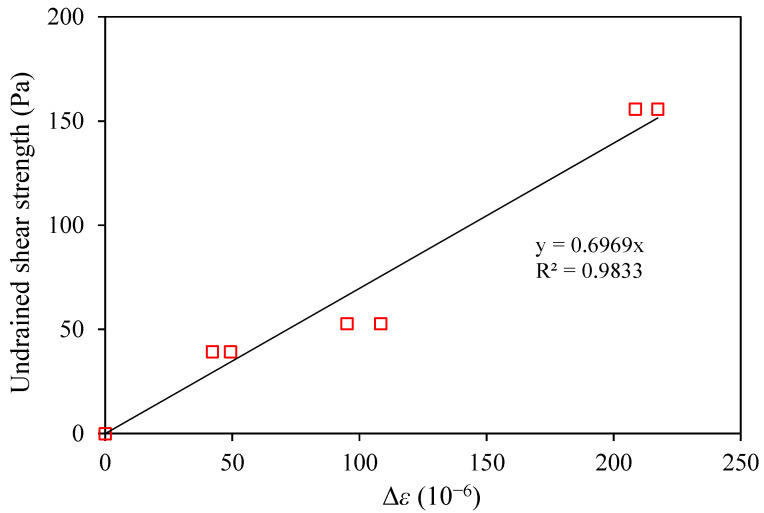
Calibration result.

Figure 4 shows typical strain distributions along the OFDR coin sensor during the calibration tests. Owing to the OFDR technology being a fully distributed optical fiber sensing technology, the ODFR interrogator can detect the strain changes along the entire optical fiber cable, including the jumping cable that connects the main sensing part of the sensor to the interrogator. The effective sensing range of the OFDR coin sensor used for the calibration was 7.3~7.8 m where the coins were located with a spacing of 50 mm on the optical fiber. It should be noted that only the strain changes within the effective sensing range were used for the undrained shear strength measurement. Each peak of the strain distribution within the effective sensing range represents the location of a coin. Due to the interaction between the coin and surrounding soil, there was a local increase and decrease in strain in the optical fiber above and below each coin. As the calibration aimed to find the coefficient *k* between the reference shear strength and average strain change in Equation (6), the interaction could be simply considered by taking the average strain. Figure 5 shows the strain changes measured by the OFDR coin sensor in HKMD slurry with the water contents of 110%, 150%, and 200%. A softening behavior can be observed from the pullup test in the HKMD slurry with the water content of 110%. The peak value might be attributed to the soil structure. It should be noted that each pulldown test was conducted after the pullup test. Therefore, no softening behavior was observed during the pulldown test since the soil structure had been degraded after the pullup test. However, no such softening behavior was observed from the pullup tests in the HKMD slurry with higher water content, indicating that soil structure can be hardly formed in the HKMD slurry if the water content is greater than 150%. There was no significant difference between the measured data for pulling up and pulling down if the residual points were selected for comparison. Besides, some spikes can be observed from measured data, which might be attributed to some unexpected disturbance to the optical fiber. Figure 6 shows the values of reference undrained shear strength of HKMD at different water contents. As expected, the undrained shear strength decreases with the increase in water content. It should be noted that the reference undrained shear strength presented here refers to the residual strength measured using an extendable hand-held vane tester. Therefore, the residual values from the OFDR coin sensor were used in this study. Comparing the measured strain from the OFDR coin sensor and the reference undrained shear strength, k=0.7 Pa/με with the correlation coefficient of 0.98 can be determined using linear regression, as shown in Figure 7. Given that the optical fibers used in this study have a diameter of 1.8 mm and elastic modulus of 72 GPa, and the coins have a diameter of 30 mm and spacing of 50 mm, the factor α=0.018 can be determined, which indicated that the real shear surface is not a cylindrical shape. It should be noted that the factor α may vary in different soils. Further study is needed to quantify the value of the factor α for different conditions.

**Figure 8:**
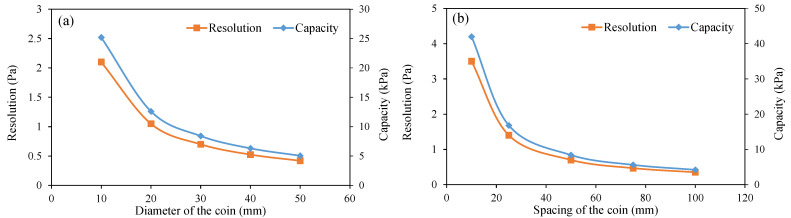
Resolution and capacity of the OFDR coin sensors with different coin diameters (**a**) and spacings (**b**).

For the current configuration, the sensitivity of the OFDR coin sensor is 0.7 Pa/με. Considering the OFDR technology used in this study has a minimum resolution of 1 mm and measurand resolution of 1 με, the resolution of the OFDR coin sensor is 0.7 Pa. The capacity of the OFDR coin sensor is 8.5 kPa. This measurement range of undrained shear strength is suitable for very soft marine soils or sediments, which means the current design of the OFDR coin sensor and the diameter and spacing of the coins are appropriate. Therefore, the diameter and spacing of the coins of the OFDR coin sensor were not changed in this study. Figure 8 shows the theoretical values of the resolution and capacity of the OFDR coin sensors with different coin diameters and spacings without correcting the shape of the shear surface. In addition, the resolution and capacity of the OFDR coin sensor also depend on the diameter and elastic modulus of the optic fiber.

## 5. Small Physical Model Test

**Figure 9:**
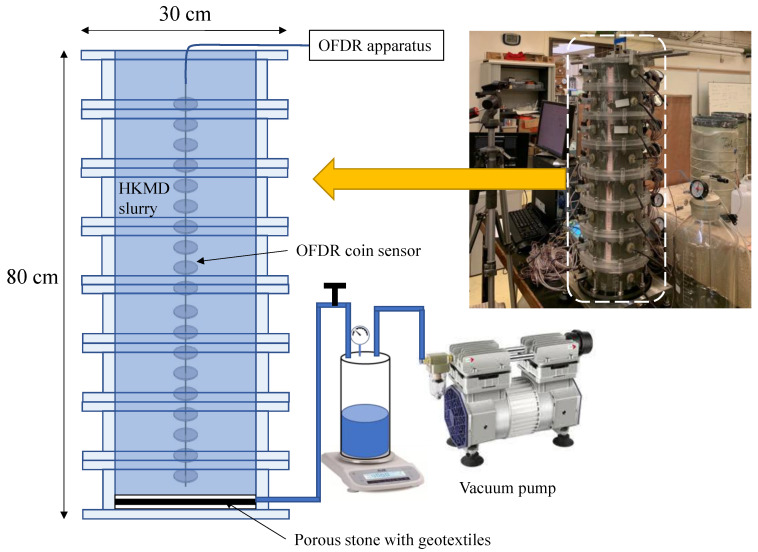
Schematic diagram of the small physical model test.

The developed OFDR coin sensor was applied to a small physical model test of HKMD slurry subjected to a vacuum loading, as shown in Figure 9.

### 5.1. Setup and Preparation

The model test was conducted in an 80 cm high sedimentation column assembled by eight acrylic cylinder sections. Each cylinder section has a dimension of 10 cm in height and 30 cm in diameter. Two sets of rulers were attached to the inner surface of the sedimentation column to measure the settlement during the vacuum loading. A piece of porous stone sandwiched by two layers of non-woven geotextiles was placed at the bottom (0 cm height) of the sedimentation column and connected to a vacuum preloading equipment. The OFDR coin sensor was installed with the help of a frame before the filling of the HKMD slurry. The initial water content of the HKMD slurry was 218%.

In this physical model test, the position of the OFDR coin sensor was fixed, while the HKMD slurry was settling down due to the self-weight consolidation and the consolidation induced by vacuum loading. The OFDR coin sensor had upward displacement relative to the settling soil. Therefore, strain can be induced along the OFDR sensor.

### 5.2. Test Results

**Figure 10:**
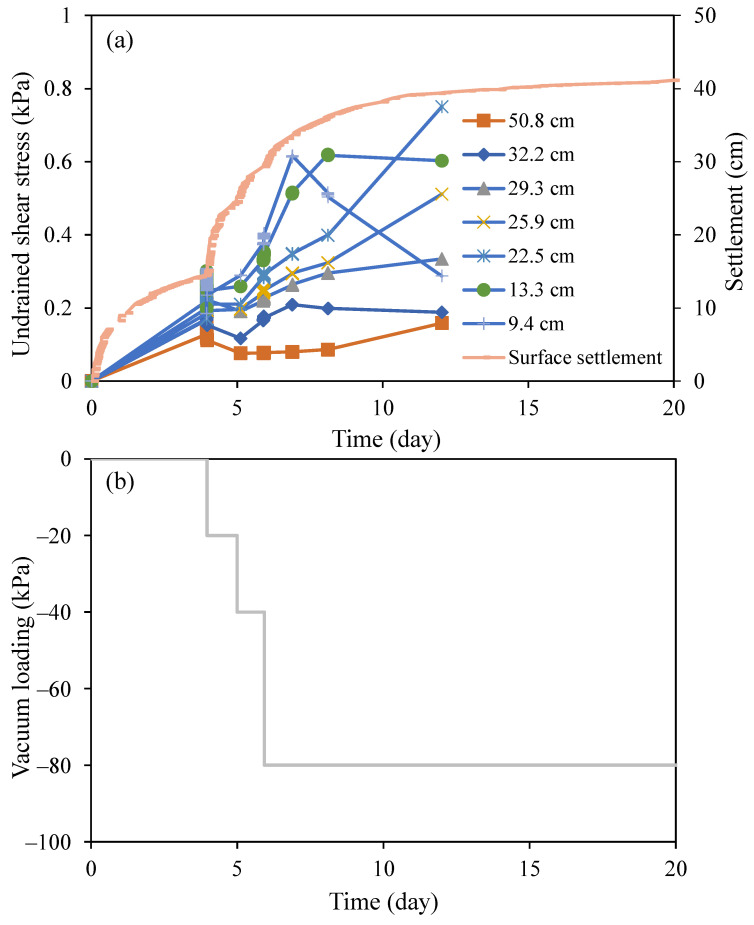
(**a**) Measured undrained shear strength and surface settlement with time; and (**b**) vacuum loading with time.

Figure 10 shows the measured undrained shear strength, surface settlement, and applied vacuum loading with time. In the first four days, no vacuum was applied, the measured settlement was mainly attributed to the sedimentation process and self-weight consolidation of the HKMD slurry. It can be seen that the undrained shear strength of around 0.2 kPa was measured by the OFDR coin sensor before the application of vacuum loading. The vacuum loadings of −20 kPa, −40 kPa, and −80 kPa were applied to the HKMD slurry from the bottom in three consecutive stages. The settlement increased significantly after Day 4 and slowly increased after Day 8. The measured undrained shear strength saw a notable increase at different heights after Day 5. However, a reduction in undrained shear strength at 9.4 cm height was observed after Day 6. This could be attributed to the fact that the relative displacement between the OFDR coin sensor and the surrounding soil near the bottom of the physical model was not sufficient to fully mobilize the shear stress, as the settlement rate decreased with time after Day 6. It can be seen from Figure 6 that a relative displacement of 40 mm should be guaranteed to obtain reliable data for undrained shear strength. In addition, a decreased settlement rate indicated a decrease in shear rate, hence reducing the measured value of undrained shear strength. Considering that the settlement rate (shear rate) after Day 12 was negligible, only the undrained shear strength measured by the OFDR coin sensor in the first 12 days is presented.

**Figure 11:**
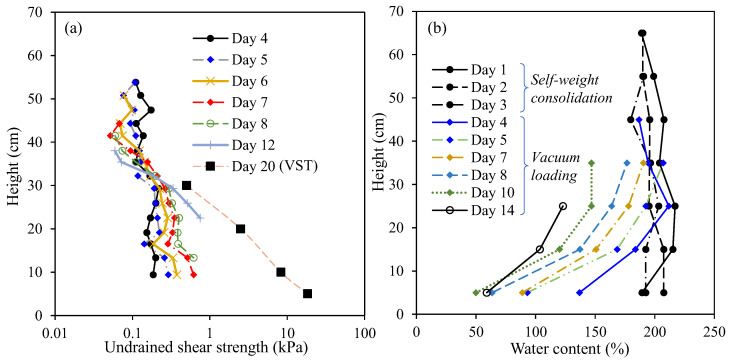
(**a**) Profiles of undrained shear strength; and (**b**) profiles of water content.

After the test, the vane shear test was conducted to measure the undrained shear strength of the soil for comparison. Figure 11a shows the undrained shear strength profiles measured by the OFDR coin sensor and the vane shear test at different times. It can be seen that values of undrained shear strength obtained from the vane shear test after Day 20 were larger than those measured by the OFDR coin sensor at Day 12. This could be attributed to the further development of undrained shear strength with the consolidation of the HKMD slurry under the vacuum loading. Figure 11b shows the water content profile at different times. The trend of undrained shear strength development agreed well with the change in water content.

The undrained shear strength measurement using the OFDR coin sensor with the fixed location is considered reliable when the surrounding soil has a significant settlement, for example during the sedimentation or self-weight consolidation. If the slurry has gained sufficient strength, shear strength measurement by pulling the OFDR coin sensor up or down is appreciated, which is presented in the next section.

## 6. Large Physical Model Test

The OFDR coin sensor was applied to a large physical model test of HKMD slurry treated by prefabricated horizontal drains (PHDs) with vacuum preloading to further demonstrate its feasibility for undrained shear strength measurement. Different from the small physical model test in which the OFDR coin sensor was fixed, the OFDR coin senor was pulled up or down in the large physical model test to measure the undrained shear strength of the HKMD slurry.

### 6.1. Setup and Preparation

**Figure 12:**
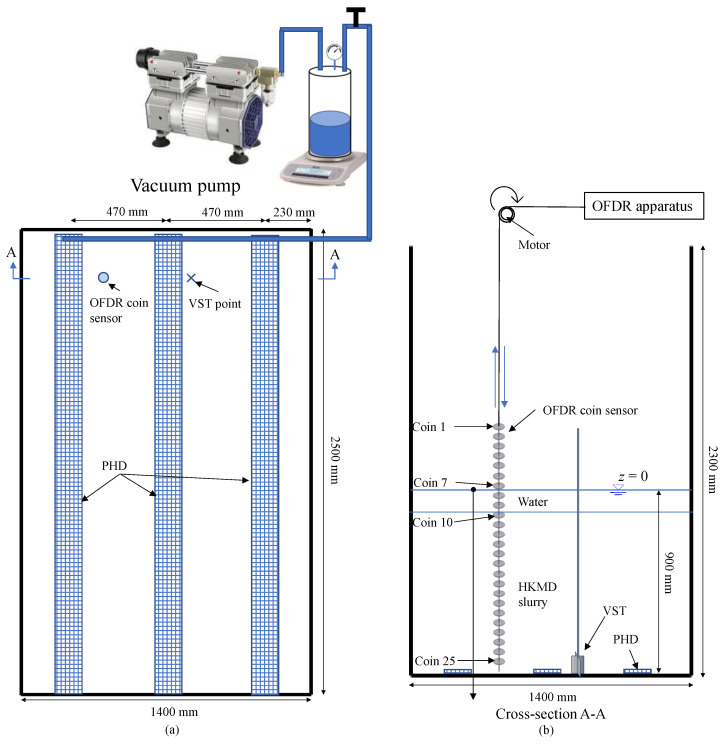
Schematic diagram of the application of the OFDR coin sensor in the large physical model test: (**a**) top view and (**b**) cross-section view.

The large physical model test of HKMD slurry treated by PHDs with vacuum preloading was conducted in a large steel tank (2500 mm in length, 1400 mm in width, 2300 mm in height). Three PHDs with the width of 100 mm and thickness of 4 mm were placed at the bottom of the model tank and connected to a vacuum pump through water pipes, as shown in Figure 12. The initial water content of the HKMD slurry was 350%. The average water content near the bottom of the large physical model was 160% after the treatment of vacuum loading for 14 days. The OFDR coin sensor with 25 coins was installed after the completion of the vacuum loading. A motor was used to pull up or down the OFDR coin sensor. For comparison, the vane shear test was also conducted at the location marked in Figure 12.

### 6.2. Test Results

**Figure 13:**
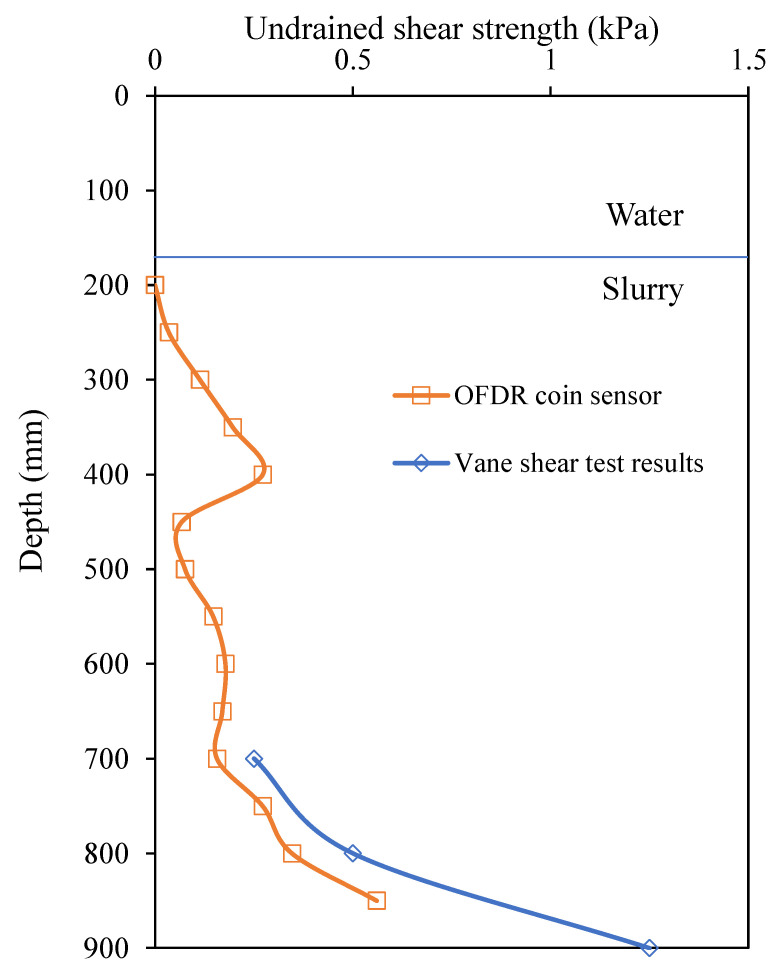
Undrained shear strength measured by the OFDR coin sensor and vane shear test.

Figure 13 shows the undrained shear strength profile of HKMD slurry along the depth measured by the OFDR coin sensor and the undrained shear strength obtained from the vane shear test. It should be noted that the surface level of the water is set as zero depth. The undrained shear strength of HKMD slurry generally increased along the depth while a peak between 300 mm to 400 mm depth was probably due to that the HKMD slurry was not uniformly mixed. Below 700 mm depth, the values of undrained shear strength measured by the OFDR coin sensor were slightly less than the vane shear test results, which might be due to the shorter drainage path of the testing location (distance to the adjacent PHD) where the vane shear test was performed. Nevertheless, the trend of the undrained shear strength profile measured by the OFDR coin sensor agreed well with the results from the vane shear test.

## 7. Discussion

**Figure 14:**
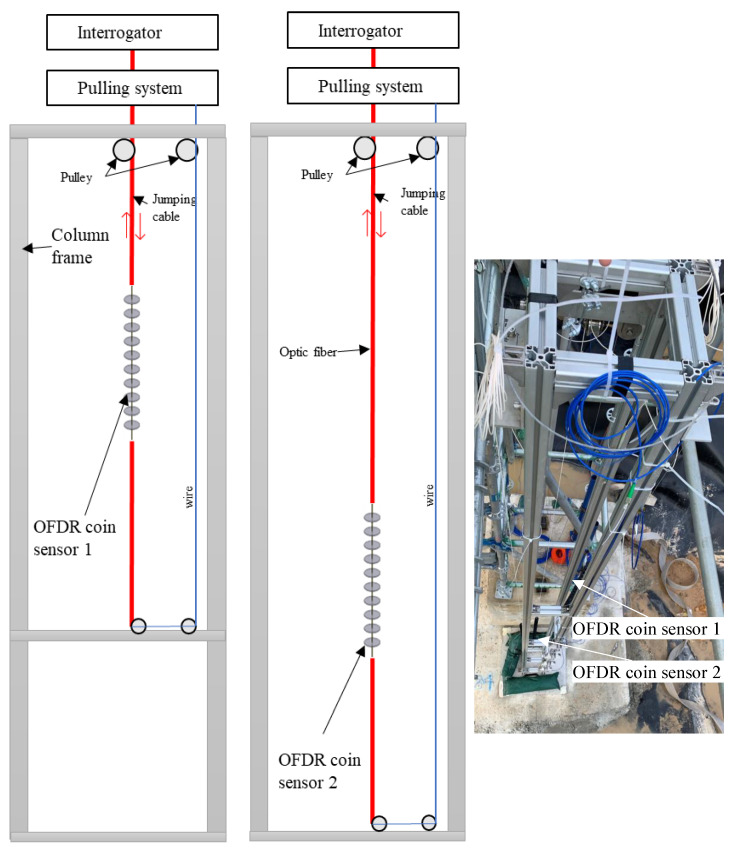
Application of OFDR coin sensor in a field trial.

The previous sections have demonstrated the feasibility of the OFDR coin sensor on undrained shear strength measurement by means of physical model tests. It is believed that the proposed sensor has the potential for in situ measurement of undrained shear strength. In fact, the OFDR coin sensor is being used in a reclamation field trial, as shown in Figure 14. However, the field application of the OFDR coin sensor and the design of the guiding frame are beyond the objectives of this paper. In addition, the field trial is ongoing. Therefore, the field testing results and detailed information on how to operate OFDR coin sensors are not presented here. Nevertheless, further studies should be conducted focusing on the installation and operation of OFDR coin sensors in different field conditions.

The OFDR coin sensor with the configuration presented in this study measures undrained shear strength from 0.7 Pa to 8.5 kPa, which is suitable for very soft marine soils or sediments. It should be noted that various measurement ranges can be achieved if optical fibers with different values of modulus or cross-section areas and coins with different diameters and/or spacings are used. However, if the OFDR coin sensor is adopted in soft soils with low water content or slightly over-consolidated soils, Equations (4) and (5) should be modified to take into account the elastic modulus of the soil within each sensing element of the sensor in order to provide the available results. Since the OFDR coin sensors are designed for application in very soft to soft sediments, the contribution of the soil modulus is not significant. The undrained shear strength measured by the OFDR coin sensor is also sensitive to the pulling rate (the shear rate to the surrounding soil). Although the OFDR coin sensors were operated at the same pulling rate in all calibration tests and the large physical model test, the effect of shear rate on measured undrained shear strength can be somewhat observed in the small physical model test. Further studies are needed to investigate the effect of shear rate and provide a reference range for practical use.

Owing to the merits of OFDR technology, the OFDR coin sensor can easily achieve a fully distributed measurement with immunity to electromagnetic interference, which guarantees good-quality signals and data, particularly for in situ application. In addition, the OFDR coin sensor can be placed horizontally so that the horizontal shear strength profile of soils can be measured. Special guiding frames should be designed for vertically or horizontally installing the OFDR coin sensor in situ and for facilitating the operation of the sensor according to the specific purpose. However, the design of such frames is beyond the focus of this paper. Apart from undrained shear strength, other soil parameters, such as friction angle and effective stress, can be roughly estimated using the proposed OFDR coin sensor if both vertical and horizontal shear stresses are measured at the same time. For example, the vertical and horizontal shear stresses can be estimated by:

(7) $$\tau_{v}=f_{v}{\sigma^{\prime }}_{h}$$

(8) $$\tau_{h}=f_{h}{\sigma^{\prime }}_{v}$$

where $f_{v}=f_{h}=\tan\varphi^{\prime }$ if the slurry is assumed to be isotropic and has no cohesion; and $\varphi^{\prime }$ is the friction angle. Knowing that ${\sigma^{\prime }}_{h}=K_{0}{\sigma^{\prime }}_{v}$ [27], $K_{0}$ can be calculated by combining Equations (7) and (8):

(9) $$K_{0}=\frac{\tau_{v}}{\tau_{h}}$$

Using Jaky’s equation $K_{0}=1-\sin\varphi^{\prime }$ [28,29], $\varphi^{\prime }$ can be calculated as:

(10) $$\varphi^{\prime }=\arcsin\left(1-\frac{\tau_{v}}{\tau_{h}}\right)$$

Therefore, the vertical and horizontal effective stresses can be calculated by combining Equations (7), (8) and (10):

(11) $${\sigma^{\prime }}_{v}=\frac{\tau_{h}}{\tan\varphi^{\prime }}$$

(12) $${\sigma^{\prime }}_{h}=\frac{\tau_{v}}{\tan\varphi^{\prime }}$$

It should be pointed out that Equations (7)–(12) are not rigorous in terms of effective parameters and can only be used for preliminary estimation of friction angle and effective stress.

## 8. Summary and Conclusions

A novel sensor with a simple design has been developed for undrained shear strength measurement for very soft to soft sediments/soils. Using OFDR sensing technology, fully distributed measurement can be easily achieved with immunity to electromagnetic interference, which guarantees good-quality signals and data, particularly for in situ application. The OFDR coin sensor has been calibrated and validated in two physical model tests with two setups. In the first physical model, a fixed setup was adopted, in which the OFDR coin sensor was fixed, while the sediments/soils were settling. The fixed setup of the sensor allows the measurement of undrained shear strength during self-weight consolidation. In the second physical model, the OFDR coin sensor was pulled up or down instead of being fixed. Both tests have demonstrated that the proposed sensor can work reliably with good resolution and accuracy. It is worth mentioning that shear stresses in both vertical and horizontal directions can be measured using OFDR coin sensors with certain guiding frames. Estimation of other soil parameters can also be made from the measured vertical and horizontal shear stresses.

## Figures and Tables

**Table 1:** Disadvantages of CPT, VST, and T-bar/ball penetrometer.

Test	Disadvantages
CPT	1. There are different correction factors for different soil types and states
	2. Effects of water pressure need to be corrected
	3. Low resolution
VST	1. Discrete measurement
	2. Not suitable for dilatant and coarse grained soils
	3. Requiring certain knowledge of soil type to correct interpretation of measured data
T-bar/ball penetrometer	1. There is a certain restriction on the size of the T-bar2. Measured data of T-bar is susceptible to bending moments3. Effects of shaft resistance need to be considered
	2. Measured data of T-bar is susceptible to bending moments
	3. Effects of shaft resistance need to be considered

**Table 2:** Specification of OFDR interrogator and optical fibers.

Item	Specification
Spatial resolution	1 mm
Measurand resolution	1 με/0.1 °C
Measurement range	±12,000 με/−200~1200 °C
Sensing length	Up to 120 m
Typo of OFDR	Coherent
Optical fiber	PVC-coated single mode silicon fiber (1.8 mm in diameter)

**Table 3:** Basic properties of HKMD.

*G_s_*	Atterberg Limits		PI (%)	pH	Loss of Ignition (%)	PSD			
	LL (%)	PL (%)				Clay (%)	Silt (%)	Sand (%)	Gravel * (%)
2.65	43.2	22.6	20.6	6.46	4.46	11.41	66.80	15.68	6.11

Note: LL refers to liquid limit, PL refers to plastic limit, and PI indicates plasticity index. * Mainly shells.

## Data Availability

Not applicable.
